# Distribution and diversity of bacterial endophytes from four *Pinus* species and their efficacy as biocontrol agents for devastating pine wood nematodes

**DOI:** 10.1038/s41598-019-48739-4

**Published:** 2019-08-28

**Authors:** Yunran Liu, Lakshmi Narayanan Ponpandian, Hoki Kim, Junhyun Jeon, Buyng Su Hwang, Sun Keun Lee, Soo-Chul Park, Hanhong Bae

**Affiliations:** 1 Department of Biotechnology, Yeungnam University, Gyeongsan, Gyeongbuk 38541 Republic of Korea; 2 Nakdonggang National Institute of Biological Resources, Sangju, Gyeongbuk 37242 Republic of Korea; 3 Division of Forest Insect Pests and Diseases, National Institute of Forest Science, Seoul, 02455 Republic of Korea; 4 Crop Biotechnology Institute, Green Bio Science & Technology, Seoul National University, Pyeongchang, Kangwon 25354 Republic of Korea

**Keywords:** Plant symbiosis, Pathogens

## Abstract

In this study, we isolated a total of 238 culturable putative bacterial endophytes from four *Pinus* species (*Pinus densiflora*, *P*. *koraiensis*, *P*. *rigida*, and *P*. *thunbergii*) across 18 sampling sites in Korea. The samples were cultured in de Man Rogosa Sharpe and humic acid-vitamin agar media. These selective media were used to isolate lactic acid bacteria and *Actinobacteria*, respectively. Analysis using 16S ribosomal DNA sequencing grouped the isolated putative bacterial endophytes into 107 operational taxonomic units (OTUs) belonging to 48 genera. *Gamma*-*proteobacteria* were the most abundant bacteria in each sampling site and three tissues (needle, stem and root). The highest OTU richness and diversity indices were observed in the roots, followed by stem and needle tissues. Total metabolites extracted from three isolates (two isolates of *Escherichia coli* and *Serratia marcescens*) showed significant nematicidal activity against the pine wood nematode (*Bursaphelenchus xylophilus*). Our findings demonstrated the potential use of bacterial endophytes from pine trees as alternative biocontrol agents against pine wood nematodes.

## Introduction

Endophytes have received significant research interest and show great potential as biocontrol agents (BCAs)^1–5^. In recent decades, emerging technologies have increasingly relied on the utilization of endophytes as natural and nontoxic sources of pesticides and fertilizers^6^. The broad applications of BEs make them commercially, economically, and scientifically important^7^. Endophytes, including bacteria and fungi, produce bioactive compounds by exploiting the conditions of their specific habitat (i.e. internal living tissues of plants). Intimate and permanent associations between endophytes and host plants can be formed without causing havoc to plant tissues. Accordingly, decades of research has demonstrated that endophytes can not only promote plant growth, but also exert protective effects against abiotic and biotic stresses^8^.

Some studies have focused on endophytic *Actinobacteria* because of their ability to produce metabolites with diverse functions^9–11^. Lactic acid bacteria (LAB), phylum *Firmicutes*, are widely used in various industrial applications as starters for food fermentation, probiotics, and BCA^12^. However, studies on endophytic LAB are limited, whereas a large number of LAB was isolated from withered leaves or plant tissues damaged by insects^13,14^. Some researchers have been unable to isolate endophytic LAB from standing live crops^15–17^. LAB are present in low numbers in the natural plant environment^18^.

Chemical methods for controlling plant parasitic nematodes have been extensively applied because of their non-selectivity. However, despite their effectiveness and availability, chemical nematicides have been reconsidered due to the disadvantages, such as high risk for environmental hazard^10,19,20^. Avermectin extracted from *Streptomyces avermitilis*, an *Actinobacteria* species, exhibits significant nematicidal activity^21^. The genus *Streptomyces* exerts nematicidal activity against parasitic nematodes^22,23^. Currently, avermectin and avermectin derivatives are used as trunk-injection agents to control the pine wood nematode (PWN, *Bursaphelenchus xylophilus*)^24,25^. PWN, the causal agent of pine wilt disease (PWD), has been initially reported in Canada and USA, infects trees across Japan, China, eastern Asian, and western Europe^25,26^. In addition, PWN caused serious damage in Korea^24,27^ where *Pinus desiflora*, *P*. *koraiensis*, and *P*. *thunbergii* are natural hosts of PWN, whereas *P*. *rigida*, a native species found in North America, is resistant^24,28^. Biocontrol against PWN using endophytes is not yet fully developed, but an interrelationship between nematodes and their associated bacteria is evident^29^. Therefore, BEs exhibit potential use as BCAs against PWN.

We hypothesize that the characterized putative BEs might be used to control for PWN. We investigated the distribution and diversity of putative BEs from four *Pinus* species grown under multiple ecological conditions across Korea. Endophyte community significantly differs among species, sampling site, and tissues of pine trees. We isolated large numbers of putative BEs that do not belong to *Actinobacteria* and LAB. We also screened the isolated putative BEs for nematicidal activity against PWN. Screening results showed that *Escherichia coli* and *Serratia marcescens* exhibited significant nematicidal activity and can thus potentially be used as BCAs against PWN. Here, we tested the hypothesis that the hosts may possess endophytes that can be a practical and effective choice as pest control agents.

## Results

### Isolation and identification of putative bacterial endophytes

Putative BEs were isolated from four *Pinus* species across 18 sampling sites in Korea (Supplementary Table S1). HV medium was used to isolate endophytic *Actinobacteria*. The analysis identified 116 endophytic isolates belonging to 74 OTUs and 26 genera (Supplementary Table S2). However, only 24 *Actinobacteria* corresponding to 12 OTUs were isolated. Other bacteria included 92 isolates representing 62 OTUs.

We attempted to isolate endophytic LAB using MRS medium. Culturing identified 131 isolates belonging to 54 OTUs and 33 genera (Supplementary Table S2). Only three isolates of LAB belonging to 2 OTUs were isolated from the root of *P*. *densiflora* in Jejudo (Pd 2), namely, *Lactococcus lactis* subsp. *lactis*, *Leuconostoc mesenteroides*, and *Leuconostoc* sp.

Taken together, we identified a total of 238 bacterial isolates belonging to 107 OTUs and 48 genera by culturing samples in MRS and HV media. Most OTUs showed more than 99% similarity with reference strains.

### Diversity, distribution, and relative abundance of putative endophytes

**Figure 1:**
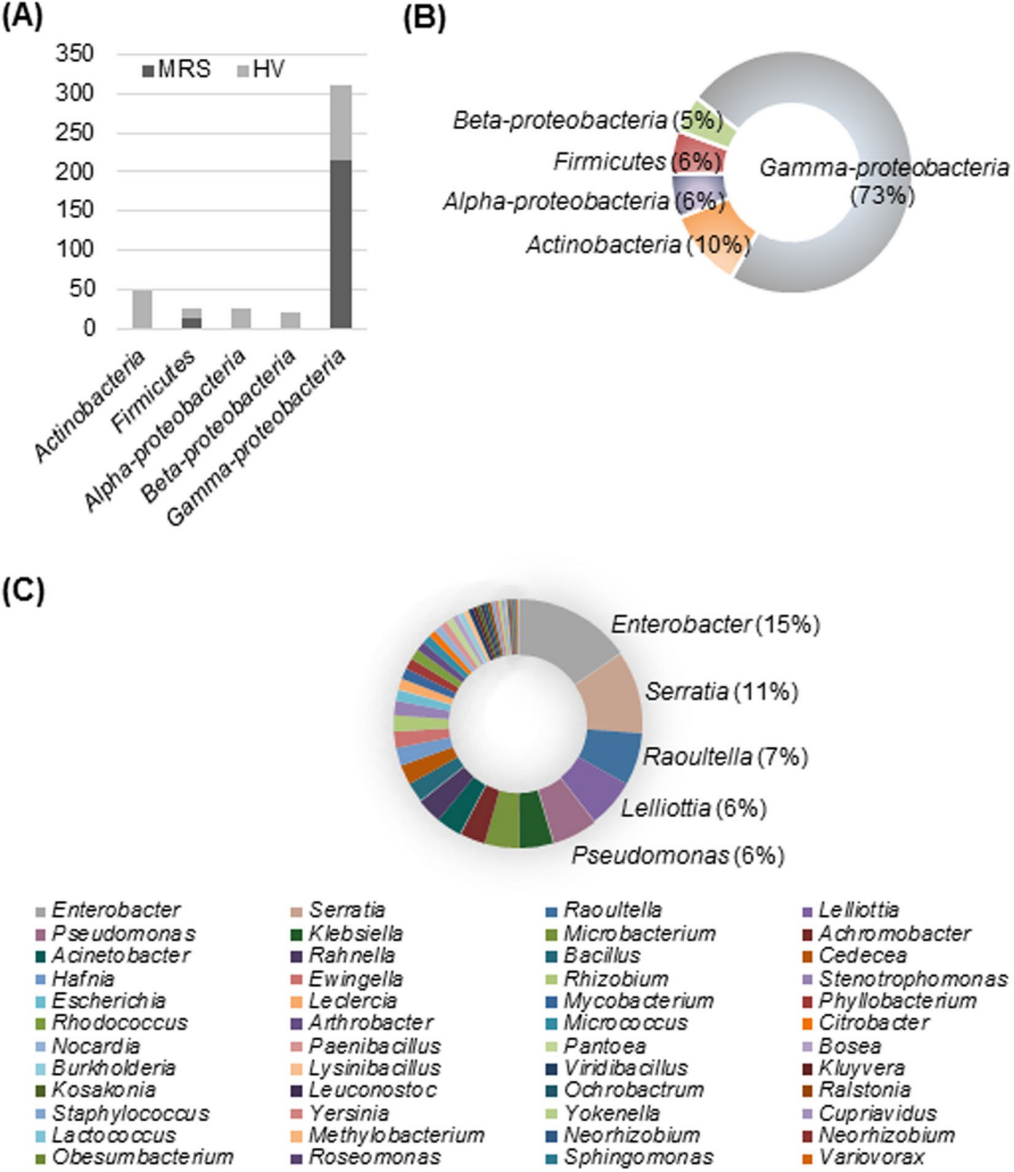
Distribution and relative abundances of putative bacterial endophytes isolated from four *Pinus* species in Korea. (**A**) Number of endophytes isolated using MRS and HV media. (**B**) Pie chart showing the relative abundances of endophytes at the phylum level. (**C**) Pie chart showing the relative abundances of endophytes at the genus level.

Phylogenetic analysis of 238 bacterial isolates revealed the relationship between the different species of BEs (Supplementary Fig. S1). The distribution of isolates showed significant differences between the MRS and HV culture media (Fig. 1). The majority of isolates that were culturable in HV media belonged to the phyla *Actinobacteria*, *Firmicutes*, and *Proteobacteria* (class: *Alpha*-, *Beta*-, and *Gamma*-*proteobacteria*) (Fig. 1A). On the other hand, the majority of isolates that were culturable in MRS belonged to *Firmicutes* and *Gamma*-*proteobacteria*. All representative isolates were clustered into the following three phyla: *Actinobacteria*, *Firmicutes* (including LAB), and *Proteobacteria* (three classes: *Alpha*-, *Beta*-, and *Gamma*-*proteobacteria*). *Gamma*-*proteobacteria* (73%) constituted the majority of the isolates, whereas *Beta*-*proteobacteria* (5%) were the least represented (Fig. 1B). Other, such as *Actinobacteria*, accounted for 10% of all isolates. Isolates belonging to *Alpha*-*proteobacteria* (6%) and *Firmicutes* (6%) showed similar relative abundance.

**Figure 2:**
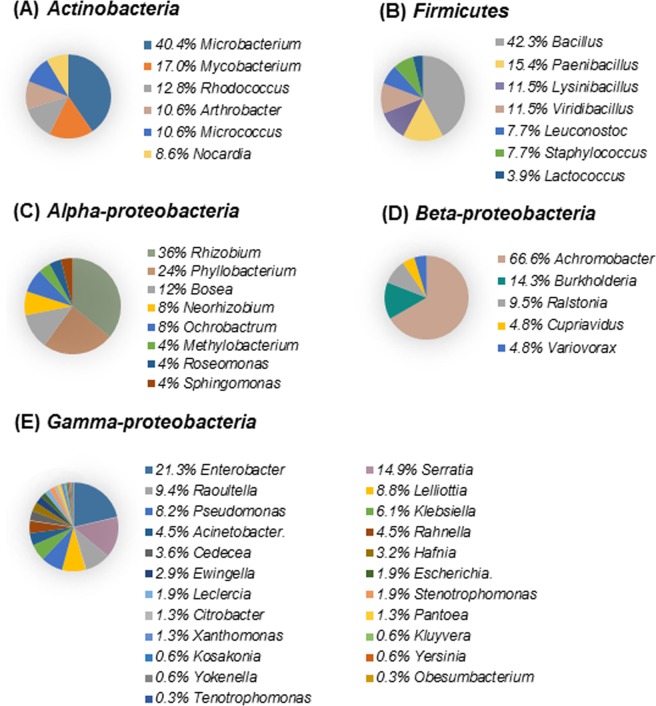
Pie charts represent the relative abundance of each genus of putative bacterial endophytes isolated from four *Pinus* species in Korea. (**A**) *Actinobacteria*. (**B**) *Firmicutes*. (**C**) *Alpha-proteobacteria*. (**D**) *Beta*-*proteobacteria*. (**E**) *Gamma*-*proteobacteria*.

The relative abundance of the 48 genera was as follows (Fig. 1C): *Enterobacter* (15%), *Serratia* (11%), *Raoultella* (7%), *Lelliottia* (6%), *Pseudomonas* (6%), *Microbacterium* (4%), and *Klebsiella* (4%). Forty-one genera comprised less than 3% of the isolates. *Gamma*-*proteobacteria* were the most abundant class (phylum) representing 23 genera, whereas *Actinobacteria*, *Firmicutes*, *Alpha*-*proteobacteria*, and *Beta*-*proteobacteria* were represented by 5, 7, 8, and 5 genera, respectively (Fig. 2).

**Figure 3:**
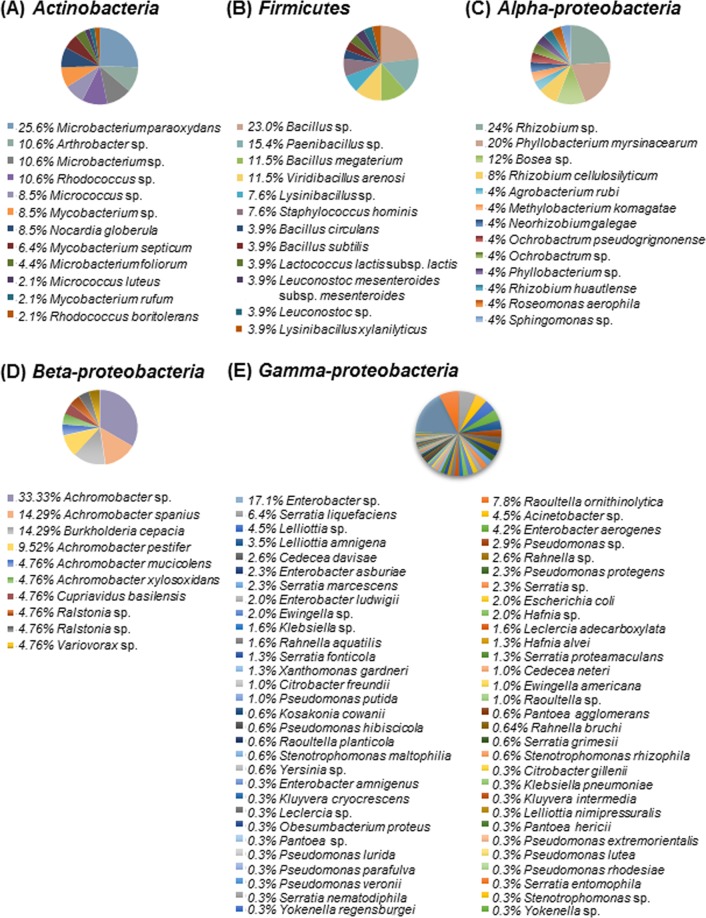
Pie charts represent the relative abundances of isolates in each phylum (class) of putative bacterial endophytes isolated from four *Pinus* species in Korea. (**A**) *Actinobacteria*. (**B**) *Firmicutes*. (**C**) *Alpha*-*proteobacteria*. (**D**) *Beta-proteobacteria*. (**E**) *Gamma*-*proteobacteria*.

At the OTU level, *Achromobacter* sp. was the most abundant (33.3%, 7 isolates) in *Beta*-*proteobacteria*, which comprise a total of 10 OTUs with 21 isolates (Fig. 3). *Bacillus* sp. was the most abundant (23.0%, 6 isolates) in *Firmicutes* (12 OTUs with 26 isolates). *Microbacterium paraoxydans* was the most abundant (25.6%, 12 isolates) in *Actinobacteria*, which consist of 12 OTUs with 47 isolates. *Rhizobium* sp. (24.0%, 6 isolates) was the most abundant in *Alpha*-*proteobacteria*, which consist of 13 OTUs with 25 isolates. *Enterobacter* sp. (17.1%, 53 isolates) comprised the majority of *Gamma*-*proteobacteria*, which comprise 60 OTUs with 311 isolates.

### Distribution of putative bacterial endophytes in different tissues of pine trees

**Figure 4:**
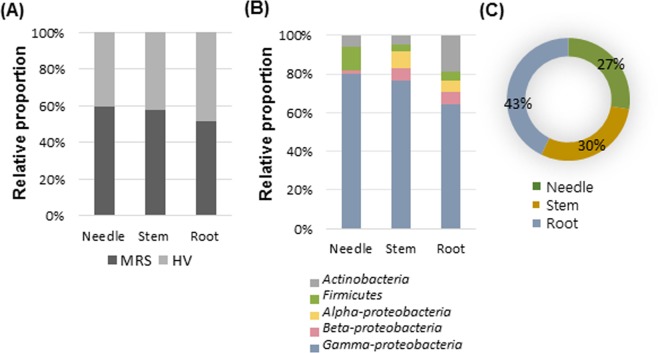
Relative abundances of putative bacterial endophytes in three different tissues (needle, stem, and root) of four *Pinus* species in Korea. (**A**) Relative proportion of endophytes isolated using MRS and HV media in three different tissues. (**B**) Relative proportion of endophytes at the phylum (class) level in three different tissues. (**C**) Pie chart showing the relative proportion of endophytes in three different tissues. Pearson chi-square test (χ^2^) was conducted to compare the endophyte composition between three different tissues (***P < 0.001).

Furthermore, we analysed the biological diversity and distribution of BEs in three different tissues (needle, stem, and root) of four pine tree species. The largest proportion of BEs was isolated from root tissues using HV media, whereas the largest proportion of BEs isolated from needle tissues using MRS media (Fig. 4A). Regarding the relative proportion of bacterial phyla/class, *Gamma*-*proteobacteria* accounted for the largest proportion of class in each tissue, especially found in the needle (Fig. 4B). *Firmicutes* (14.1%) and *Beta*-*proteobacteria* (1.7%) were the most and least abundant, respectively. The highest percentage of *Actinobacteria* was found in the root (18.9%). Similarly, the large proportion of *Alpha*-*proteobacteria* (8.8%) was observed in the stem. Overall, the composition of root and needle endophytes seems to be significantly different, while there was no difference in other comparisons based on Pearson chi-square test (***P < 0.001). In summary, the largest number of isolates was isolated from root (43%), followed by stem (30%) and needle tissues (27%) (Fig. 4C).

**Figure 5:**
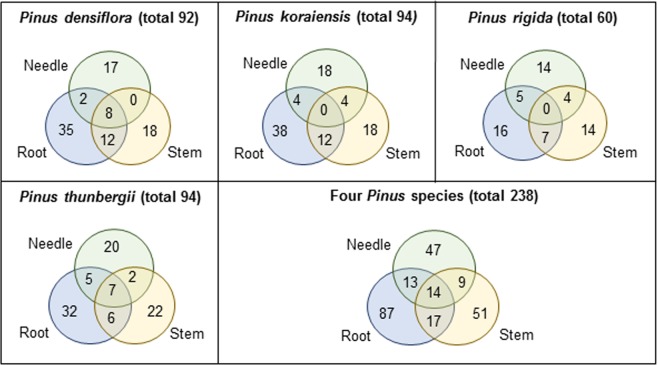
Venn diagram showing the distribution of putative bacterial endophytes in three different tissues (needle, stem, and root) of four *Pinus* species in Korea. The distribution of 238 isolates was described in three tissues of four different *Pinus* species.

**Figure 6:**
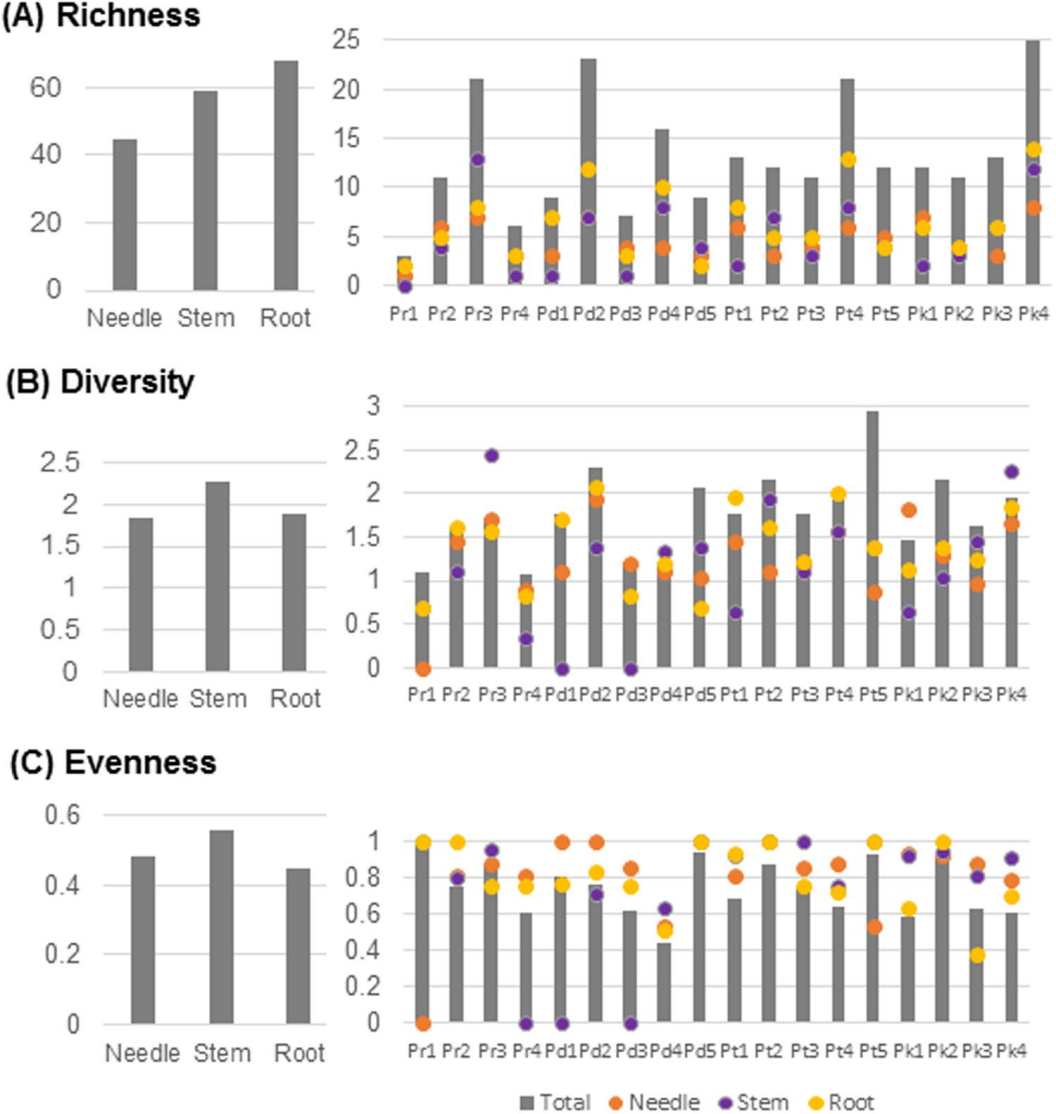
Operational taxonomic unit (OTU) diversity of putative bacterial endophytes in three different tissues (needle, stem, and root) of four *Pinus* species across 18 sampling sites in Korea. (**A**) Bar graph representing the overall OTU richness in three different tissues and across the different sampling sites. (**B**) Bar graph representing the overall OTU diversity (Shannon’s index, H′) in three different tissues and across the different sampling sites. (**C**) Bar graph representing the overall OTU evenness in three different tissues across the different sampling sites. Dots indicate the richness, diversity, and evenness of different tissues in each site.

Figure 5 describes the distribution of 238 isolates in three tissues of four different *Pinus* species. We identified 92, 94, 60, and 94 isolates in *P*. *densiflora*, *P*. *koraiensis*, *P*. *rigida*, and *P*. *thunbergii*, respectively. No identical BEs were isolated from all tissues in *P*. *koraiensis* and *P*. *rigida*. No identical BEs were identified in both the needle and stem tissues of *P*. *densiflora*. Overall, root tissues exclusively harboured 29 OTUs (87 isolates; the highest OTU richness), followed by 20 OTUs (51 isolates) in stem tissues, and 13 OTUs (47 isolates) in the needle. Some isolates were simultaneously present in two tissues, while 14 isolates were found in all three tissues. Subsequently, the biological diversity of BEs was analysed for each tissue. The highest OTU richness were found in the root (68 OTUs, followed by stem (59 OTUs), and needle tissues (45 OUTs Fig. 6A). Nevertheless, the bacterial OTUs in the root tissues were unevenly distributed, lowest diversity of OTU (H′ = 1.885) and evenness (E = 0.446) found in root. The highest level of diversity and evenness was found in stem tissues (H′ = 2.278, E = 0.978; Fig. 6B,C).

### Distribution of putative endophytes in different sampling sites

Samples isolated from *P*. *densiflora* and *P*. *thunbergii* were collected from 5 sampling sites, named Pd 1 to 5 and Pt 1 to 5, respectively. Samples from *P*. *koraiensis* and *P*. *rigida* were collected from 4 sampling sites, designated Pk 1 to 4 and Pr 1 to 4, respectively.

There were significant differences in the distribution of OTUs among the 18 sampling sites, indicating high OTU richness. The highest OTU richness was found in Pk 4 (25 OTUs), and more than 20 OTUs were identified in Pr 3, Pd 2, and Pt 4. Pr 1 showed the lowest OTU richness among the sampling sites (3 OTUs) (Fig. 6A). Likewise, biological diversity was analyzed using Shannon index (H′). The highest H′ value was found in Pt 5 (H′ = 2.945), The lowest index was detected in Pr 1 (H′ = 1.098) (Fig. 6B). Furthermore, the highest OTU evenness (E) values were observed in Pr 1 (E = 1) and Pd 5 (E = 0.943), while the lowest evenness was detected in Pd 4 (E = 0.438; Fig. 6C).

**Figure 7:**
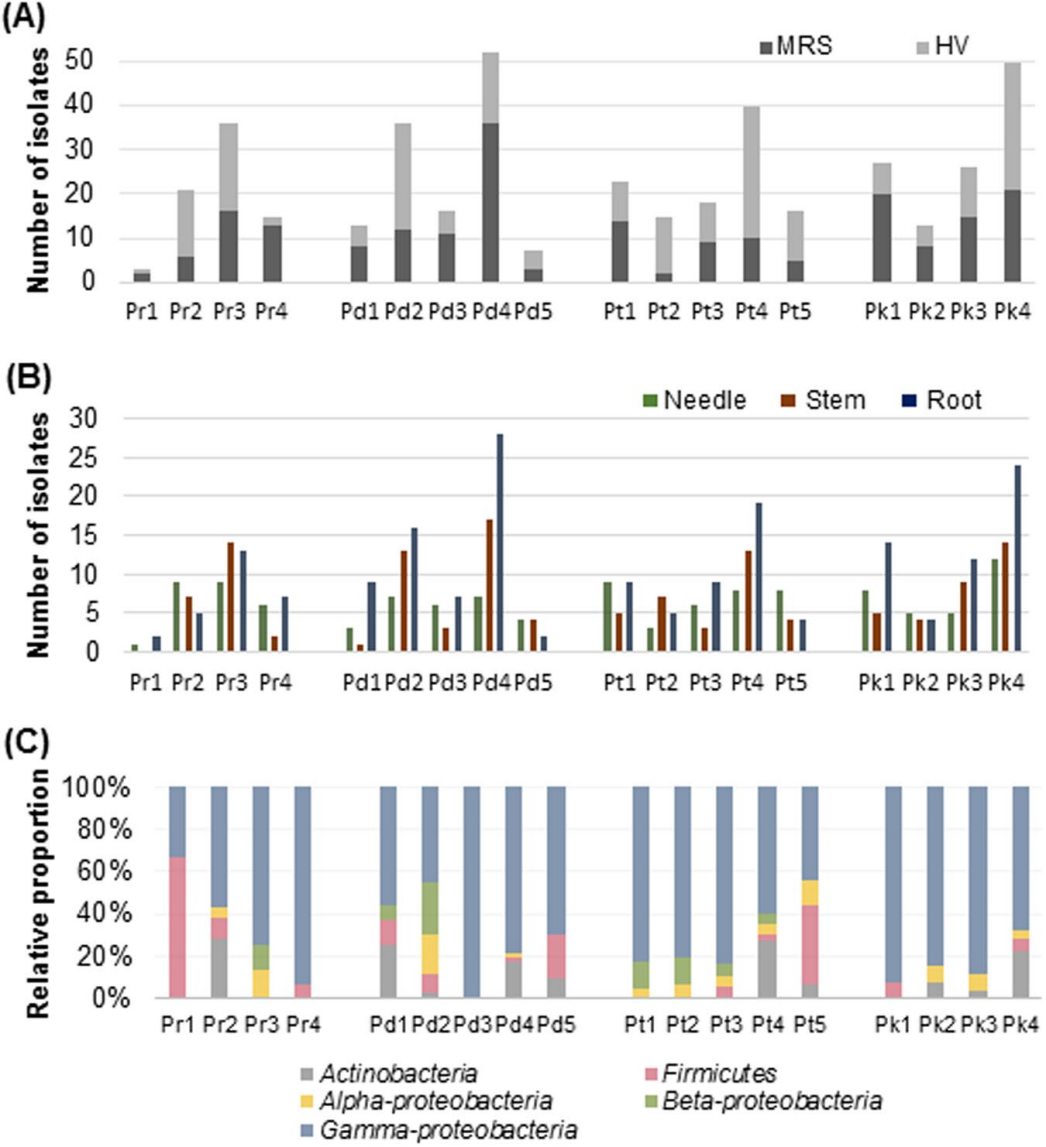
Numbers and relative proportions of putative bacterial endophytes isolated from three different tissues (needle, stem, and root) of four *Pinus* species across 18 sampling sites in Korea. (**A**) Bar graph representing the total number of isolates using MRS and HV media across sampling sites. (**B**) Bar graph representing total number of isolates across sampling sites for each different tissue. (**C**) Bar graph representing the relative proportion of isolates at the phylum (class) level.

More BEs were isolated from Pr 2, Pr 3, Pd 2, Pd 5, Pt 2, Pt 4, Pt 5, and Pk 4 using HV media than using MRS media (Fig. 7A). The distribution of BEs isolated from different tissues in each sampling site was analyzed (Fig. 7B). In the majority of sampling sites, the largest number of BEs was isolated from root tissue, followed by stem and needle tissues. The largest number of BEs was from needle tissues in Pr 2, Pt 5, and Pk 2, while the largest number of isolates was from stem tissues in Pr 3 and Pt 2. *Gamma*-*proteobacteria* accounted for the largest proportion of isolates obtained across all sampling sites, except for Pr 1 (Fig. 7C). The largest proportion of *Gamma*-*proteobacteria* (100%) was found in Pd 3, while the largest proportion of *Firmicutes* (66.7%) was found in Pr 1. The highest proportion of *Actinobacteria* was found in Pr 2 (28.6%) and Pt 4 (27.5%). The highest proportion of *Alpha*-*proteobacteria* and *Beta*-*proteobacteria* were observed in Pd 2.

### Nematicidal activity of total metabolites against pine wood nematode

**Figure 8:**
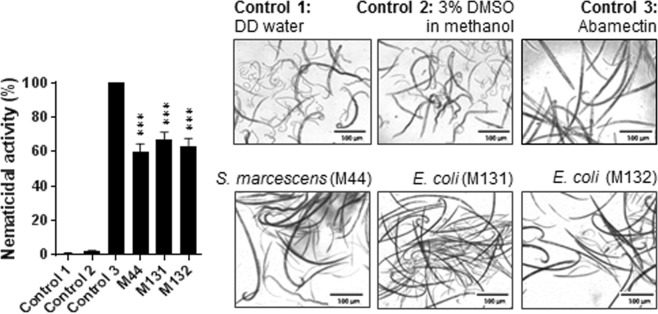
Nematicidal activity of ethyl acetate extracts isolated from the three selected putative bacterial endophytes. Nematodes (*Bursaphelenchus xylophilus*) were monitored after 12 h of treatment. Bar graph shows the percentage of dead nematodes at 1,000 ppm treatment. Data are the mean ± standard error of four biological replicates with six technical replicates (*n* = 24). Control 1 = double distilled (DD) water. Control 2 = 3% DMSO in 100% methanol (solvent control). Control 3 = Abamectin (1,000 ppm). *Serratia marcescens* (M44), *Escherichia coli* (M131), and *Escherichia coli* (M132). Significant difference at ***P < 0.001 *vs*. control 2. Scale bars = 100 μm.

Total metabolites extracted from BEs using ethyl acetate (EtOAc) were used to test nematicidal activity against PWN. Eight-day-old adult nematodes were used for the screening. Linearization of PWN was observed after 12 h of treatment. Primary screening identified 17 isolates that showed strong nematicidal activity, corresponding to greater than 50% mortality of nematodes after 12 h of treatment (data not shown). Among 17 isolates, 76% were *Proteobacteria*, while others belonged to 3 *Firmicutes* and 1 *Actinobacteria*. In particular, we identified 3 isolates from 2 different species, *Escherichia coli* (M131, 67% mortality; M132, 63% mortality) and *Serratia marcescens* (M44, 60% mortality) (Fig. 8). Three isolates showed significantly higher nematicidal activity compared to control 1 and 2 at a level of *P* < 0.001.

## Discussion

PWD is a common destructive disease in pine tree typically caused by PWN. To prevent progression of the disease, nematicidal compounds such as abamectin and avermectin have been extensively used. These nematicidal compounds were originally isolated from *Streptomyces avermitilis*^30,31^, suggesting that environmental bacteria could be a good source of a myriad of bioactive metabolites. Previous studies have demonstrated that *Actinobacteria* and LAB have nematicidal activity^30–32^. Thus, the goal of the present study was to isolate culturable putatively endophytic *Actinobacteria* and LAB from different tissues (needle, stem and root) of pine trees using MRS and HV media in search for possible biocontrol agents for PWN.

We identified a total of 24 (12 OTUs) and 3 (3 OTUs) isolates of culturable *Actinobacteria* and LAB, respectively. The majority of the isolated BEs (211 isolates, 92 OTUs) did not belong to *Actinobacteria* and LAB. In addition, we identified 219 unique isolates representing 60 OTUs using MRS/HV media. These findings expanded the set of the culturable BEs in pine trees isolated using different culture media.

*Actinobacteria* were predominantly isolated from various sources such as air, water, and plants^33^. Here, the majority of the 24 *Actinobacteria* were found in root tissues (72%) corroborating the results in the previous studies^34,35^. Species belonging to *Actinobacteria* are the predominant microbiota found in plant roots attributing to the close associations between root and soil bacteria. Moreover, epidermal breakage caused by differentiation of branch roots might facilitate the entry and colonization of *Actinobacteria* into root tissues. In cereal crops, more number of *Actinobacteria* exist in the rhizosphere^36–38^. *Actinobacteria* are well-known to be in association with the rhizosphere and in colonizing plant internal tissues^39,40^. Among the 6 identified genera of *Actinobacteria* in this study, *Microbacterium* has been shown to be the dominant genus. Interestingly, we isolated *Microbacterium paraoxydans*, which has been reported to promote plant growth^41^.

LAB are present inside living plants because of their acid-tolerance and anaerobic properties^18^. LAB have been isolated from plant surfaces and damaged plant tissues^13,16^. Similarly, many isolates have been cultivated from stem tissues of lodgepole pine trees (*Pinus contorta)*^42^. The functions of LAB in plants and the exact reasons for their abundance in damaged plants are still not clear. Under suitable conditions, it has been shown that lactic fermentation occurs spontaneously in harvested vegetables or fruits^43^, in which LAB play a significant role. Previous studies have demonstrated that damaged plants can secrete specific chemical signals, including organic acids such as oxaloacetate and malic acid that can attract beneficial bacteria to mediate plant defense responses^44,45^. A similar mechanism is likely to occur for LAB-mediated plant protection. LAB can produce antagonistic compounds and provide an acidic environment that can protect plants from pathogenic microorganisms^43^. In this study, three LAB OTUs were isolated from the roots of *P*. *densiflora* obtained from Jejudo. Among 18 sampling sites, the Jejudo sampling site showed the lowest soil pH (5.3) condition and high organic contents (50.6 g/kg) which possibly explain the occurrence of these LAB isolates in association with pine trees in this area.

Microbes in the environment are attracted towards fruits and flowers^46^, by plant substance, and dispersed to other tissues^47^. The migration of BEs involves whole plant from the root to needle^48^, thereby posing a possibility of bacterial migration to and from any of the three tissues (needle, stem and root). Our results showed that 14 BE isolates were shared among all three tissues. Root and stem tissues were found to share a high number of BEs (17 isolates), whereas most BEs were exclusively found in a single tissue type. Our findings indicated that all BE phyla were distributed among the stem and root. The highest proportions of *Firmicutes* and *Gamma*-*proteobacteria* were found in the needle. *Actinobacteria* were more abundant in the root than in the needle and stem, exemplifying its reported high abundance in soil^49^. In addition, the proportion of *Alpha*-*proteobacteria* was higher in the stem than in the root. Most of the BEs in this study were isolated using HV medium (pH 7.2), however a portion of *Gamma*-*proteobacteria* (50%) and *Firmicutes* (3%) were isolated under acidic medium (MRS, pH 6.2) suggesting that these isolated BEs might be tolerant to acidic condition and can uptake carbon and nitrogen from humic acid. In general, the diversity and relative abundance of BEs are influenced by various factors, including the host plants, environment, and cultivation method^34,50^.

Many endophytes exert preventive effects against plant diseases and can colonize plants for long-term and stable periods. *Bacillus pumilus* and *B*. *cereus* isolated from pine trees in China have been shown to exhibit strong nematicidal activity against PWN^51^. Furthermore, a novel bacterial strain has been identified that could secrete serine/neutral proteases that degrade the integument of nematode, which cause leakage of nematode contents resulting to the death of the parasitic nematodes^52^. In search for alternative sources of nematicidal compounds for PWN, we have tested secondary metabolites from the isolated pine tree BEs. We have found two strains of *Escherichia coli*, and *Serratia marcescens* that exhibited significant nematicidal activity. In a previous study, *S*. *marcescens* isolated from infected *Pinus pinaster* showed strong nematicidal activity of its total metabolite^53^.

In summary, endophytic *Actinobacteria* and LAB have been isolated from pine trees albeit with low abundance compared to other phyla and species, respectively. Putative endophytic bacterial community from pine trees noticeably differs in all three tissues, and most of the bacteria can be distinguished as tissue-specific and differ from one sampling site to another. The isolated *Escherichia coli* strains and *Serratia marcescens* could potentially be used as biocontrol agents for PWN. These efforts provide a better understanding of the composition of pine tree bacterial endophytes and also an empirical baseline to explore beneficial BEs for biocontrol of PWN. Further studies investigating the mechanisms of action of BEs’ metabolites as well as the identification and purification of active compound are necessary to develop strategies for controlling PWN.

## Methods

### Sample preparation

Pine tree samples (needle, stem and root tissues) were collected from four *Pinus* species (*P*. *densiflora*, *P*. *koraiensis*, *P*. *rigida* and *P*. *thunbergii*) across 18 sampling sites in Korea from June to August 2016 (Supplementary Table S1). Pine tree samples were collected from five (*P*. *densiflora* and *P*. *thunbergii*) to four (*P*. *koraiensis* and *P*. *rigida*) sampling sites. Tissue samples were collected from six trees (biological replicates) from each sampling site. Each tissue sample was collected from two opposite sides of the tree. Young needle samples (2-years old) were collected using sterilized blades. Stem samples were collected at one meter above the ground using a sterilized increment borer. Tertiary root tissues were collected without uprooting at a depth of 10 to 25 cm below the ground. Samples were individually placed in clean zip bags and stored at 4 °C until further analysis.

All tissue samples (needle, stem and root tissues) were dissected and weighed (1 g) separately. The samples were washed thrice in sterilized reverse osmosis (RO) treated water and sonicated for 20 s to remove loam and organic substance^54–56^. Samples were rinsed in sterile 0.1% Tween-20 for 5 min, 70% ethanol for 5 min, and 4% sodium hypochlorite (NaOCl) solution for 5 min. Next, samples were washed ten times in RO water to remove the chemicals and further soaked in sterile 10% (w/v) sodium bicarbonate (NaHCO_3_) for 10 min to inhibit the growth of fungal endophytes. The samples were then washed ten times in RO water again. To make sure that the sample surface was sterile, samples were rolled on tryptic soy agar (TSA) and nutrient agar (NA) plates (Thermo Fisher Scientific, Waltham, MA, USA), and the Petri dishes were monitored for up to 15 days at 28 °C. Each sample was ground in 10 ml of sterile phosphate buffer (10 mM, pH 7.2), shaken at 110 rpm for 30 min at 28 °C, and subsequently stored at 4 °C.

### Isolation of putatively endophytic Actinobacteria and lactic acid bacteria

Phosphate buffer was serially diluted for five to ten dilutions. Each dilution (500 µL) was added to 5 mL of HV broth (MB Cell, LA, CA, USA)^57^ with shaking at 150 rpm for up to 2 weeks at 30 °C. The liquid culture (100 µL) was plated on HV agar and incubated at 30 °C for up to 3 weeks. After sub-culturing, pure colonies on the TSA plates were differentiated based on shape, size, colour, texture, form, height and edge. Finally, individual colonies were stored in glycerol stocks.

The procedure for isolation of putatively endophytic LAB was identical to the method employed for isolating endophytic *Actinobacteria* but using MRS medium (Thermo Fisher Scientific). Each dilution (500 µL) of phosphate buffer was add to 5 mL of MRS broth and shaken at 150 rpm at 30 °C for up to 1 week. After spreading the turbid liquid medium on MRS agar plates, the plates were incubated at 30 °C for up to 1 week for the selection of the different colonies.

### Molecular identification

The 16S ribosomal DNA (rDNA) region was amplified from all BEs using universal primers (27 F 5′-AGAGTTTGATCCTGGCTCAG-3′; 1492 R 5′-GGTTACCTTGTTACGACTT-3′) according to the Bionics protocol (Seoul, Korea)^58^. Amplified sequences were analysed using Geneious version v. 10.1.3 software (Biomatters, Auckland, New Zealand)^59,60^ and validated via NCBI BLAST search (Supplementary Dataset 1). MEGA v. 7.0 was used to build the phylogenetic tree using the neighbour-joining method and a bootstrap test with 1,000 replications (http://www.megasoftware.net)^61^.

### Extraction of total metabolites from putative bacterial endophytes

Total metabolites were isolated from tryptic soya broth (TSB) cultures using ethyl acetate (EtOAc)^62,63^. Endophytes were cultured at 30 °C overnight in TSB with shaking at 150 rpm. TSB cultures were added with an equal volume of EtOAc, sonicated for 1 h, and shaken at 120 rpm overnight. The mixture was allowed to stand for 2 h. The top clear phase was transferred to a new flask. EtOAc was evaporated using a rotary evaporator at 50 °C to concentrate the crude extracts. The crude extracts were dissolved in 3% dimethyl sulfoxide (DMSO) in 100% methanol.

### Data analysis

Relative abundance was estimated as the percentage of the number of isolates belonging to a particular OTU or phylum divided by the total number of isolates. OTU richness (S) represents the number of OTU recovered from a specific sampling site or tree tissue. OTU evenness (E) was calculated using the following equation: E = H/H_max_, where H_max_ is the maximum value of Shannon’s diversity index (H). H was calculated using the following equation: H = −Σ (P*i* × In P*i*), where P*i* is the relative proportion of OTU *i* in a sampling site or tree tissue^64^. Graphs were generated and statistical analysis of two-way analysis of variance (ANOVA) (Tukey) was performed to test for significant differences between treatments using GraphPad Prism 7 (La Jolla, CA, USA). Pearson chi-square test (χ^2^) was performed using R programming (https://www.R-project.org/). OTUs were used to calculate for OTU richness, diversity and the related graphs.

### Nematicidal activity test

PWN was obtained from National Institute of Forest Science (Seoul, Korea). PWNs were fed with the fungus *Botrytis cinerea*, and the plate was incubated at 25 °C in the dark for 8 days^65^. PWN was harvested using Baermann funnel technique^66^. PWN was suspended in sterile water, counted, and separated in 96-well polystyrene plates (100 nematodes in 90 µL of water). Crude extracts (0.1 mg in 10 µL, final concentration = 1,000 ppm) were added to the wells of a 96-well polystyrene plate, and the plate was incubated at 25 °C in dark for 12 h. The following treatments were used as controls: control 1, double distilled (DD) water; control 2, 3% DMSO in methanol; positive control, abamectin at 1,000 ppm. Dead nematodes were observed under a microscope and counted. The percentages of dead nematodes were calculated for descriptive analysis. According to the Schneider-Orelli formula, the mortality of nematodes can be corrected by ignoring the mortality in the control^67^: Corrected mortality (%) = (mortality % in treatment − mortality % in negative control)/(1 − mortality % in negative control).

## Supplementary information

Supplementary Figure and Tables
