# Supplementary material for: Distribution and diversity of bacterial endophytes from four Pinus species and their efficacy as biocontrol agents for devastating pine wood nematodes
Source: Sci Rep. 2019 Aug 28;9:12461. doi: 10.1038/s41598-019-48739-4 (PMC6713764; doi:10.1038/s41598-019-48739-4)
Supplement: Supplementary file 1 — Supplementary Figure and Tables [file 41598_2019_48739_MOESM1_ESM.pdf]

Distribution and diversity of bacterial endophytes from four *Pinus* species and their efficacy as biocontrol agents for devastating pine wood nematodes

Yunran Liu[1], Lakshmi Narayanan Ponpandian[1], Hoki Kim[1], Junhyun Jeon[1], Buyng Su Hwang[2], Sun Keun Lee[3], Soo-Chul Park[4], Hanhong Bae[1,*]

[1] Department of Biotechnology, Yeungnam University, Gyeongsan, Gyeongbuk 38541, Republic of Korea

[2] Nakdonggang National Institute of Biological Resources, Sangju, Gyeongbuk 37242, Republic of Korea

[3] Division of Forest Insect Pests and Diseases, National Institute of Forest Science, Seoul 02455, Republic of Korea

[4] Crop Biotechnology Institute, Green Bio Science & Technology, Seoul National University, Pyeongchang, Kangwon 25354, Republic of Korea

[*]**Correspondence:** Hanhong Bae

Email: hanhongbae@ynu.ac.kr

Phone: 8253-810-3031 (office), Fax: 8253-810-4769,

Yunran Liu and Lakshmi Narayanan Ponpandian contributed equally to this work.

Keywords: bacterial endophytes, operational taxonomic units (OTUs), nematicidal activity, pine wilt disease, pine wood nematode (*Bursaphelenchus xylophilus*)

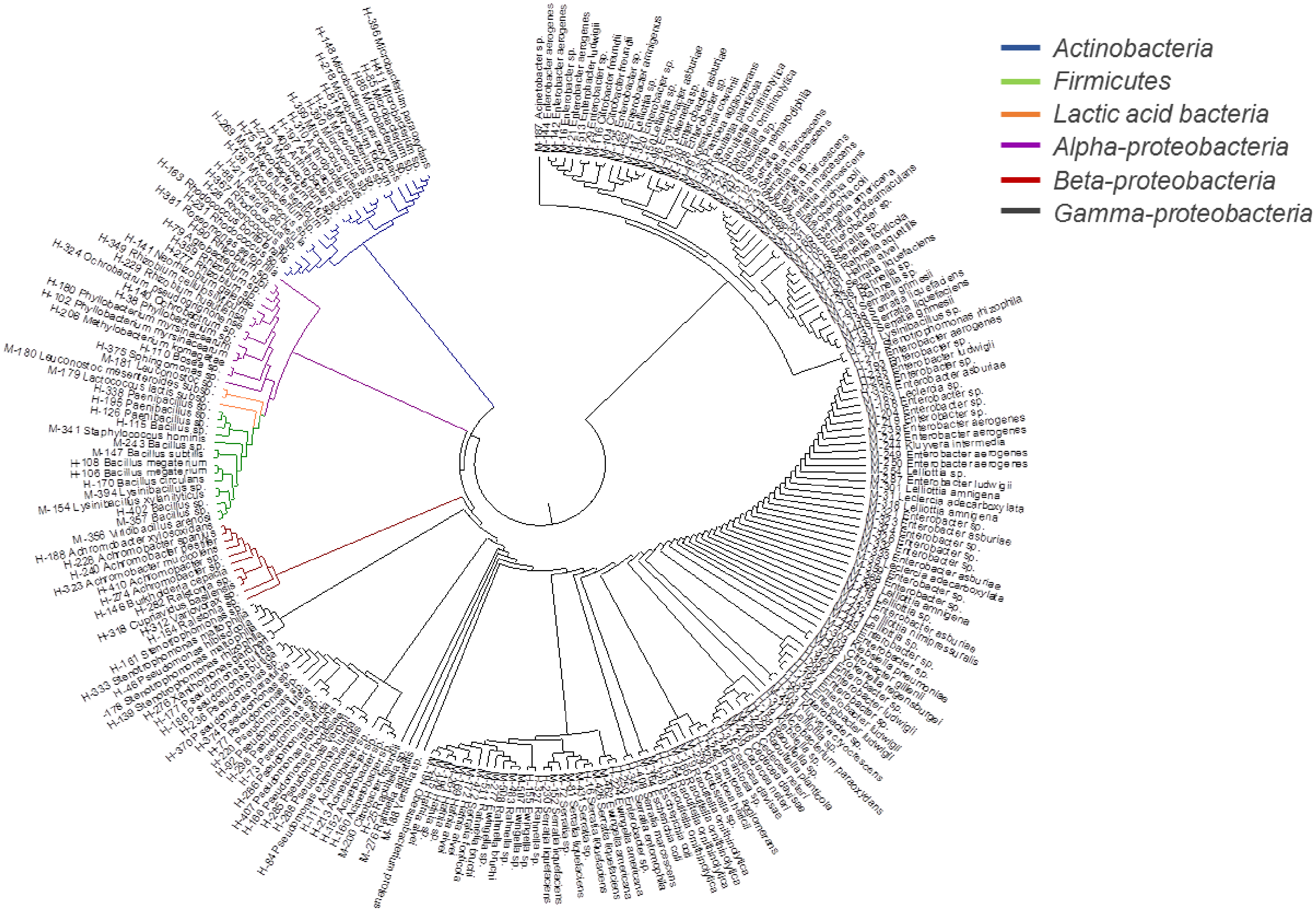

Supplementary Figure S1. Phylogenetic tree showing the placement of different isolates with reference strains based on 16S ribosomal DNA sequence. The phylogenetic tree was produced by neighbor-joining methods with 1,000 bootstrap replicates using ClustlW and Mega7.

Supplementary Table S1. Overview of the geographic location and its meta-data of 18 sampling sites in Korea.

Pinus sp.	Region	Symbol	Estimated tree age	Available silicic acid (mg/kg)	Electrical conductivity	Latitude	Longitude	pH	Available phosphate (mg/kg)	Organic matter (g/kg)	Mean Temp. (℃) 2016	Mean rainfall (mm) 2016	Mg (cmol+/kg)	Ca	K	Altitude (meter)
Pinus rigida	Anseong	Pr 1	45	123.2	0.4	37.074458	127.11192	5.9	252.4	20.8	25.5	284	1.7	4.2	0.7	70
	Seosan	Pr 2	45	75.9	1.1	36.8906971	126.449172	6.1	336.6	22.3	25.2	295.6	2.4	6.6	1.1	60
	Jungeup	Pr 3	45	7.9	0.2	35.5521138	127.019157	5.3	892.7	30.4	27.1	205.1	1.9	5.8	1	240
	Yungyang	Pr 4	43	96.2	0.1	36.6061179	129.088533	6.1	251.2	21.4	23	323.9	2.8	7.4	0.8	250
Pinus densiflora	Jungeup	Pd 1	50	7.9	0.2	35.5565492	126.98662	5.3	892.7	30.4	27.1	205.1	1.9	5.8	1	310
	Jejudo	Pd 2	50	0	1.7	33.3737599	126.471605	5.3	591.7	50.6	27.4	98.6	1.8	4.6	1.2	1030
	Hoengseong	Pd 3	45	48.9	0.8	37.5098629	128.160384	5.7	547.2	30.2	25.7	360.1	1.1	3.7	0.8	540
	Bonghoa	Pd 4	35	11.2	0.4	37.0054026	128.941595	6.1	571.7	27	25.9	416.5	1.5	5.5	0.9	700
	Yangpung	Pd 5	50	248.8	0.6	37.6118298	127.44368	6	299.6	24.4	25.4	385	1.1	5.1	0.5	300
Pinus thunbergii	Pohang	Pt 1	40	116.3	0.5	36.1205372	129.411768	5.6	153.3	22.1	25.9	113.2	2.5	6.9	0.5	1010
	Jejudo	Pt 2	45	0.1	38.7	33.4729632	126.500568	5.3	464	55.7	27.4	98.6	2	5.5	12	30
	Seosan	Pt 3	50	75.9	1.1	36.8766539	126.38679	6.1	336.6	22.3	25.2	295.6	2.4	6.6	1.1	40
	Gangneung	Pt 4	35	49.6	0.3	37.916556	128.810361	5.6	667.1	25.3	24.1	424.2	1.2	3.3	0.6	10
	Yeosu	Pt 5	45	81	0.8	34.7087622	127.760002	6.1	526.2	31.9	25.3	157.8	1.6	6.4	0.8	30
Pinus koraiensis	Hongchun	Pk 1	30	138.4	0.4	37.8073347	127.929426	5.8	217.9	21.7	25.3	429.4	1.1	4.2	0.4	470
	Jecheon	Pk 2	30	70.8	0.2	37.1883303	127.983829	5.8	320.5	25.5	24.1	419.7	1.8	5.6	0.7	320
	Chuncheon	Pk 3	35	30.1	0.6	37.9058734	127.931245	6.2	302.6	30.2	25.2	617.1	1.6	4.1	0.6	250
	Pocheon	Pk 4	50	72.4	0.4	37.9654135	127.112321	5.7	237.3	21.3	25.1	366.3	0.9	3.9	0.9	120

Supplementary Table S2. Identification of putative bacterial endophytes isolated from four *Pinus* species in Korea based on 16S ribosomal DNA sequence. H and M stand for HV and MRS media, respectively.

Isolate	Reference strain	with Accession No.	Phylum	Similarity
H16	*Serratia liquefaciens*	CP006252.1	*Gammaproteobacteria*	99%
H19	*Klebsiella* sp.	DQ831003.2	*Gammaproteobacteria*	100%
H21	*Rhodococcus* sp.	JX564535.1	*Actinobacteria*	99%
H25	*Raoultella* sp.	KU362619.1	*Gammaproteobacteria*	97%
H28	*Rhodococcus* sp.	AY864650.1	*Actinobacteria*	99%
H31	*Microbacterium paraoxydans*	LT629770.1	*Actinobacteria*	99%
H35	*Cedecea davisae*	AB682275.1	*Gammaproteobacteria*	99%
H38	*Phyllobacterium* sp.	KM253051.1	*Alphaproteobacteria*	99%
H46	*Pseudomonas hibiscicola*	KP208626.1	*Gammaproteobacteria*	99%
H73	*Pseudomonas* sp.	KM253253.1	*Gammaproteobacteria*	99%
H75	*Mycobacterium rufum*	KU341399.1	*Actinobacteria*	100%
H77	*Pseudomonas* sp.	MF144536.1	*Gammaproteobacteria*	99%
H79	*Agrobacterium rubi*	KR708890.1	*Alphaproteobacteria*	99%
H81	*Microbacterium* sp.	LC133735.2	*Actinobacteria*	99%
H84	*Pseudomonas extremorientalis*	LT629708.1	*Gammaproteobacteria*	99%
H85	*Microbacterium* sp.	JX566572.1	*Actinobacteria*	99%
H86	*Microbacterium* sp.	KF261555.1	*Actinobacteria*	99%
H88	*Nocardia globerula*	NR_104795.1	*Actinobacteria*	99%
H90	*Rhizobium* sp.	KM253034.1	*Alphaproteobacteria*	99%
H92	*Pseudomonas lutea*	AB495128.1	*Gammaproteobacteria*	99%
H93	*Enterobacter* sp.	AJ489826.1	*Gammaproteobacteria*	99%
H102	*Phyllobacterium myrsinacearum*	AB681130.1	*Alphaproteobacteria*	99%
H103	*Kluyvera cryocrescens*	KC355278.1	*Gammaproteobacteria*	99%
H106	*Bacillus megaterium*	LT797523.1	*Firmicutes*	99%
H108	*Bacillus megaterium*	CP018874.1	*Firmicutes*	99%
H110	*Bosea* sp.	LC133745.2	*Alphaproteobacteria*	99%
H111	*Acinetobacter* sp.	KC128833.1	*Gammaproteobacteria*	99%
H113	*Raoultella ornithinolytica*	CP021329.1	*Gammaproteobacteria*	99%
H115	*Bacillus* sp.	AB043843.1	*Firmicutes*	99%
H126	*Paenibacillus* sp.	AB746849.1	*Firmicutes*	98%
H132	*Enterobacter ludwigii*	KC355281.1	*Gammaproteobacteria*	99%

H136	*Mycobacterium* sp.	CP015596.1	*Actinobacteria*	99%
H137	*Stenotrophomonas rhizophila*	KY474340.1	*Gammaproteobacteria*	99%
H139	*Stenotrophomonas rhizophila*	GU186108.1	*Gammaproteobacteria*	99%
H140	*Ochrobactrum* sp.	LC150701.1	*Alphaproteobacteria*	99%
H141	*Neorhizobium galegae*	HG938355.1	*Alphaproteobacteria*	97%
H146	*Burkholderia cepacia*	KX055886.1	*Betaproteobacteria*	99%
H148	*Microbacterium paraoxydans*	KY425786.1	*Actinobacteria*	99%
H152	*Acinetobacter* sp.	KY228963.1	*Gammaproteobacteria*	99%
H154	*Ralstonia* sp.	KY906998.1	*Betaproteobacteria*	96%
H155	*Ewingella* sp.	AB766012.1	*Gammaproteobacteria*	99%
H158	*Raoultella* sp.	KX130779.2	*Gammaproteobacteria*	99%
H160	*Acinetobacter* sp.	JN605359.1	*Gammaproteobacteria*	100%
H161	*Stenotrophomonas* sp.	KT580577.1	*Gammaproteobacteria*	99%
H163	*Rhodococcus boritolerans*	AB288061.1	*Actinobacteria*	99%
H164	*Raoultella ornithinolytica*	CP017802.1	*Gammaproteobacteria*	99%
H166	*Pseudomonas rhodesiae*	KU321261.1	*Gammaproteobacteria*	99%
H170	*Bacillus circulans*	KF475811.1	*Firmicutes*	99%
H177	*Pseudomonas putida*	LT707061.1	*Gammaproteobacteria*	99%
H178	*Stenotrophomonas maltophilia*	JN084034.1	*Gammaproteobacteria*	98%
H180	*Phyllobacterium myrsinacearum*	KJ147062.1	*Alphaproteobacteria*	99%
H186	*Pseudomonas putida*	KX817279.1	*Gammaproteobacteria*	99%
H188	*Achromobacter xylosoxidans*	KP967463.1	*Betaproteobacteria*	99%
H192	*Enterobacter ludwigii*	KC355280.1	*Gammaproteobacteria*	99%
H193	*Enterobacter* sp.	KF803325.1	*Gammaproteobacteria*	99%
H195	*Paenibacillus* sp.	JX402418.1	*Firmicutes*	99%
H197	*Arthrobacter* sp.	KY511276.1	*Actinobacteria*	99%
H206	*Methylobacterium komagatae*	AB703238.1	*Alphaproteobacteria*	99%
H213	*Acinetobacter* sp.	KC858868.1	*Gammaproteobacteria*	99%
H217	*Enterobacter aerogenes*	CP014748.1	*Gammaproteobacteria*	99%
H218	*Microbacterium foliorum*	CP019892.1	*Actinobacteria*	99%
H220	*Pseudomonas* sp.	JF274936.1	*Gammaproteobacteria*	99%
H224	*Ewingella americana*	HE585222.1	*Gammaproteobacteria*	99%
H228	*Achromobacter spanius*	LN890048.1	*Betaproteobacteria*	99%
H229	*Rhizobium huautlense*	KC355318.1	*Alphaproteobacteria*	99%
H230	*Serratia liquefaciens*	CP014017.1	*Gammaproteobacteria*	99%
H231	*Rhodococcus* sp.	CP017299.1	*Actinobacteria*	99%

H235	*Enterobacter ludwigii*	KM077046.1	*Gammaproteobacteria*	99%
H236	*Pseudomonas* sp.	KF312476.1	*Gammaproteobacteria*	100%
H238	*Micrococcus* sp.	KP345967.1	*Actinobacteria*	98%
H240	*Achromobacter pestifer*	HG324051.1	*Betaproteobacteria*	99%
H242	*Pantoea agglomerans*	CP014129.1	*Gammaproteobacteria*	99%
H248	*Pantoea* sp.	KF479585.1	*Gammaproteobacteria*	99%
H250	*Enterobacter* sp.	JX067653.1	*Gammaproteobacteria*	99%
H263	*Enterobacter* sp.	AB673456.1	*Gammaproteobacteria*	99%
H268	*Pseudomonas lurida*	AJ581999.1	*Gammaproteobacteria*	100%
H269	*Mycobacterium septicum*	NR_042916.1	*Actinobacteria*	99%
H274	*Achromobacter* sp.	LC133607.2	*Betaproteobacteria*	99%
H276	*Xanthomonas gardneri*	CP018731.1	*Gammaproteobacteria*	99%
H277	*Rhizobium* sp.	AM403584.1	*Alphaproteobacteria*	100%
H279	*Mycobacterium* sp.	AB613819.1	*Actinobacteria*	99%
H282	*Ralstonia* sp.	KT183537.1	*Betaproteobacteria*	99%
H285	*Pseudomonas veronii*	LT599583.1	*Gammaproteobacteria*	100%
H286	*Pseudomonas putida*	KF010369.1	*Gammaproteobacteria*	100%
H297	*Enterobacter* sp.	LC007924.1	*Gammaproteobacteria*	100%
H298	*Pseudomonas* sp.	JF740045.1	*Gammaproteobacteria*	99%
H303	*Klebsiella pneumoniae*	FN689724.1	*Gammaproteobacteria*	99%
H310	*Arthrobacter* sp.	EU036697.1	*Actinobacteria*	99%
H312	*Variovorax* sp.	EU934231.1	*Betaproteobacteria*	99%
H318	*Cupriavidus basilensis*	AY047217.1	*Betaproteobacteria*	99%
H323	*Achromobacter mucicolens*	KT716268.1	*Betaproteobacteria*	99%
H324	*Ochrobactrum pseudogrignonense*	CP015776.1	*Alphaproteobacteria*	99%
H326	*Pantoea hericii*	KU189725.1	*Gammaproteobacteria*	99%
H327	*Rahnella* sp.	LC008364.1	*Gammaproteobacteria*	99%
H329	*Enterobacter* sp.	JX566614.1	*Gammaproteobacteria*	99%
H333	*Stenotrophomonas maltophilia*	LT222224.1	*Gammaproteobacteria*	99%
H336	*Enterobacter asburiae*	CP011863.1	*Gammaproteobacteria*	99%
H338	*Paenibacillus* sp.	KF011653.1	*Firmicutes*	99%
H343	*Serratia entomophila*	NR_025338.1	*Gammaproteobacteria*	99%
H349	*Rhizobium cellulosilyticum*	JN412065.1	*Alphaproteobacteria*	99%
H350	*Enterobacter ludwigii*	EF175735.1	*Gammaproteobacteria*	99%
H359	*Rhizobium* sp.	KF465962.1	*Alphaproteobacteria*	99%
H367	*Rhodococcus* sp.	MF351723.1	*Actinobacteria*	99%

H370	*Pseudomonas parafulva*	CP009747.1	*Gammaproteobacteria*	99%
H374	*Pseudomonas* sp.	CP018743.1	*Gammaproteobacteria*	100%
H375	*Sphingomonas* sp.	FN377699.1	*Alphaproteobacteria*	99%
H381	*Roseomonas aerophila*	NR_109678.1	*Alphaproteobacteria*	99%
H390	*Micrococcus* sp.	KP345951.1	*Actinobacteria*	99%
H396	*Microbacterium paraoxydans*	KF783215.1	*Actinobacteria*	100%
H399	*Micrococcus luteus*	KT339390.1	*Actinobacteria*	99%
H402	*Bacillus* sp.	LC150681.1	*Firmicutes*	99%
H406	*Arthrobacter* sp.	JF772504.1	*Actinobacteria*	99%
H407	*Pseudomonas protegens*	CP022097.1	*Gammaproteobacteria*	100%
H408	*Serratia marcescens*	KP903466.1	*Gammaproteobacteria*	99%
H410	*Achromobacter* sp.	KY907012.1	*Betaproteobacteria*	99%
H411	*Microbacterium* sp.	KR058846.1	*Actinobacteria*	99%
M16	*Enterobacter* sp.	JQ511864.1	*Gammaproteobacteria*	99%
M19	*Raoultella ornithinolytica*	CP008886.1	*Gammaproteobacteria*	99%
M21	*Enterobacter aerogenes*	KM974656.1	*Gammaproteobacteria*	99%
M29	*Enterobacter* sp.	KT887985.1	*Gammaproteobacteria*	99%
M31	*Leclercia adecarboxylata*	KC252602.1	*Gammaproteobacteria*	99%
M35	*Leclercia adecarboxylata*	HQ242722.1	*Gammaproteobacteria*	97%
M44	*Serratia marcescens*	CP011642.1	*Gammaproteobacteria*	99%
M45	*Serratia* sp.	KP325087.1	*Gammaproteobacteria*	99%
M46	*Serratia marcescens*	KP682489.1	*Gammaproteobacteria*	99%
M49	*Kosakonia cowanii*	CP019447.1	*Gammaproteobacteria*	99%
M65	*Serratia marcescens*	KX783602.1	*Gammaproteobacteria*	99%
M72	*Serratia* sp.	JQ734475.1	*Gammaproteobacteria*	99%
M81	*Serratia liquefaciens*	KJ004486.1	*Gammaproteobacteria*	99%
M82	*Serratia liquefaciens*	KY643700.1	*Gammaproteobacteria*	99%
M86	*Serratia marcescens*	LT575490.1	*Gammaproteobacteria*	99%
M93	*Serratia grimesii*	NR_025340.1	*Gammaproteobacteria*	99%
M94	*Raoultella planticola*	EU545405.1	*Gammaproteobacteria*	99%
M96	*Enterobacter asburiae*	JQ659696.1	*Gammaproteobacteria*	99%
M104	*Citrobacter freundii*	CP012554.1	*Gammaproteobacteria*	99%
M105	*Enterobacter sp.*	HQ824845.1	*Gammaproteobacteria*	99%
M106	*Serratia proteamaculans*	KC951919.1	*Gammaproteobacteria*	99%
M112	*Serratia* sp.	KY780231.1	*Gammaproteobacteria*	99%
M115	*Serratia* sp.	KU750792.1	*Gammaproteobacteria*	99%

M116	*Citrobacter freundii*	KU570343.1	*Gammaproteobacteria*	99%
M125	*Enterobacter* sp.	KF010358.1	*Gammaproteobacteria*	99%
M126	*Rahnella aquatilis*	KJ004463.1	*Gammaproteobacteria*	99%
M128	*Enterobacter* sp.	EU855187.1	*Gammaproteobacteria*	99%
M129	*Cedecea neteri*	CP009458.1	*Gammaproteobacteria*	99%
M130	*Enterobacter asburiae*	CP007546.1	*Gammaproteobacteria*	99%
M131	*Escherichia coli*	CP018770.2	*Gammaproteobacteria*	99%
M132	*Escherichia coli*	KY678497.1	*Gammaproteobacteria*	99%
M133	*Pantoea agglomerans*	KT765839.1	*Gammaproteobacteria*	98%
M137	*Serratia grimesii*	KC167881.1	*Gammaproteobacteria*	99%
M138	*Serratia liquefaciens*	CP011303.1	*Gammaproteobacteria*	99%
M142	*Enterobacter aerogenes*	CP014029.1	*Gammaproteobacteria*	99%
M144	*Enterobacter aerogenes*	KY621520.1	*Gammaproteobacteria*	99%
M147	*Bacillus subtilis*	EU096316.1	*Firmicutes*	99%
M150	*Lelliottia* sp.	KX822678.1	*Gammaproteobacteria*	99%
M151	*Serratia nematodiphila*	KY887771.1	*Gammaproteobacteria*	99%
M152	*Serratia liquefaciens*	KU950364.1	*Gammaproteobacteria*	98%
M154	*Lysinibacillus xylanilyticus*	KY316397.1	*Firmicutes*	99%
M161	*Hafnia alvei*	CP009706.1	*Gammaproteobacteria*	99%
M163	*Leclercia* sp.	HM165189.1	*Gammaproteobacteria*	100%
M164	*Escherichia coli*	CP020048.1	*Gammaproteobacteria*	99%
M166	*Raoultella ornithinolytica*	CP013338.1	*Gammaproteobacteria*	99%
M167	*Rahnella* sp.	KF192056.1	*Gammaproteobacteria*	99%
M168	*Enterobacter* sp.	KM657475.1	*Gammaproteobacteria*	100%
M169	*Hafnia alvei*	KY940341.1	*Gammaproteobacteria*	99%
M172	*Hafnia alvei*	KC210858.1	*Gammaproteobacteria*	99%
M179	*Lactococcus lactis subsp.*	KC754747.1	*Firmicutes*	99%
M180	*Leuconostoc mesenteroides*	LC260033.1	*Firmicutes*	99%
M181	*Leuconostoc* sp.	AB690198.1	*Firmicutes*	99%
M187	*Acinetobacter* sp.	KY440055.1	*Gammaproteobacteria*	100%
M188	*Yersinia* sp.	AJ011333.1	*Gammaproteobacteria*	99%
M194	*Obesumbacterium proteus*	CP014608.1	*Gammaproteobacteria*	99%
M196	*Hafnia* sp.	AP017469.1	*Gammaproteobacteria*	99%
M200	*Citrobacter freundii*	LN854584.1	*Gammaproteobacteria*	95%
M204	*Enterobacter* sp.	HQ231920.1	*Gammaproteobacteria*	99%
M207	*Klebsiella* sp.	KU193768.1	*Gammaproteobacteria*	99%

M216	*Cedecea davisae*	KX062011.1	*Gammaproteobacteria*	99%
M219	*Enterobacter* sp.	KF582906.1	*Gammaproteobacteria*	99%
M228	*Raoultella planticola*	CP023877.1	*Gammaproteobacteria*	99%
M230	*Klebsiella* sp.	CP020657.1	*Gammaproteobacteria*	99%
M234	*Raoultella ornithinolytica*	KY317922.1	*Gammaproteobacteria*	99%
M235	*Hafnia alvei*	CP015379.1	*Gammaproteobacteria*	99%
M239	*Enterobacter aerogenes*	KU726959.1	*Gammaproteobacteria*	99%
M241	*Raoultella ornithinolytica*	KY022421.1	*Gammaproteobacteria*	99%
M242	*Enterobacter aerogenes*	CP011574.1	*Gammaproteobacteria*	99%
M243	*Bacillus* sp.	KJ726746.1	*Firmicutes*	99%
M244	*Kluyvera intermedia*	KR054969.1	*Gammaproteobacteria*	99%
M249	*Enterobacter aerogenes*	CP011539.1	*Gammaproteobacteria*	99%
M250	*Enterobacter aerogenes*	KP716694.1	*Gammaproteobacteria*	99%
M253	*Cedecea neteri*	CP009451.1	*Gammaproteobacteria*	100%
M254	*Lelliottia* sp.	KJ810589.1	*Gammaproteobacteria*	99%
M261	*Hafnia* sp.	KJ781891.1	*Gammaproteobacteria*	99%
M276	*Rahnella aquatilis*	KT958515.1	*Gammaproteobacteria*	96%
M277	*Ewingella* sp.	KT958490.1	*Gammaproteobacteria*	99%
M287	*Enterobacter ludwigii*	KR822481.1	*Gammaproteobacteria*	100%
M301	*Lelliottia amnigena*	KT986086.1	*Gammaproteobacteria*	99%
M318	*Lelliottia amnigena*	KT986089.1	*Gammaproteobacteria*	99%
M320	*Enterobacter* sp.	JQ864377.1	*Gammaproteobacteria*	99%
M321	*Enterobacter* sp.	HQ231953.1	*Gammaproteobacteria*	99%
M323	*Enterobacter asburiae*	JQ659874.1	*Gammaproteobacteria*	99%
M324	*Enterobacter* sp.	AY689045.1	*Gammaproteobacteria*	96%
M326	*Enterobacter* sp.	AY689062.1	*Gammaproteobacteria*	98%
M328	*Escherichia coli*	CP010228.1	*Gammaproteobacteria*	99%
M332	*Enterobacter* sp.	KF010360.1	*Gammaproteobacteria*	99%
M333	*Enterobacter asburiae*	KC568144.1	*Gammaproteobacteria*	99%
M338	*Rahnella* sp.	JX994143.1	*Gammaproteobacteria*	99%
M341	*Staphylococcus hominis*	KX432211.1	*Firmicutes*	99%
M356	*Viridibacillus arenosi*	KJ671467.1	*Firmicutes*	99%
M357	*Bacillus* sp.	KF956642.1	*Firmicutes*	99%
M359	*Enterobacter* sp.	KM253094.1	*Gammaproteobacteria*	100%
M368	*Enterobacter* sp.	KF010357.1	*Gammaproteobacteria*	99%
M369	*Lelliottia amnigena*	KM114915.1	*Gammaproteobacteria*	99%

M373	*Serratia marcescens*	CP013046.2	*Gammaproteobacteria*	99%
M391	*Lysinibacillus* sp.	KY774365.1	*Firmicutes*	99%
M394	*Lysinibacillus* sp.	JN247743.1	*Firmicutes*	99%
M402	*Yokenella regensburgei*	KU161317.1	*Gammaproteobacteria*	99%
M403	*Yokenella* sp.	KF874625.1	*Gammaproteobacteria*	99%
M404	*Citrobacter gillenii*	NR_041697.1	*Gammaproteobacteria*	99%
M417	*Lelliottia* sp.	KY780216.1	*Gammaproteobacteria*	99%
M419	*Lelliottia* sp.	KY780217.1	*Gammaproteobacteria*	99%
M424	*Enterobacter asburiae*	KY316493.1	*Gammaproteobacteria*	99%
M426	*Serratia liquefaciens*	KJ781948.1	*Gammaproteobacteria*	99%
M431	*Serratia* sp.	KF201712.1	*Gammaproteobacteria*	99%
M432	*Lelliottia nimipressuralis*	KT986079.1	*Gammaproteobacteria*	99%
M436	*Lelliottia* sp.	KX709881.1	*Gammaproteobacteria*	98%
M441	*Lelliottia* sp.	KM458060.1	*Gammaproteobacteria*	99%
M448	*Serratia liquefaciens*	KU999993.1	*Gammaproteobacteria*	99%
M449	*Enterobacter* sp.	JQ864388.1	*Gammaproteobacteria*	99%
M452	*Enterobacter amnigenus*	AB773290.1	*Gammaproteobacteria*	99%
M458	*Serratia fonticola*	KX257354.1	*Gammaproteobacteria*	99%
M462	*Ewingella americana*	KT031282.1	*Gammaproteobacteria*	99%
M465	*Ewingella americana*	KY126990.1	*Gammaproteobacteria*	99%
M472	*Serratia fonticola*	LT555292.1	*Gammaproteobacteria*	99%
M477	*Enterobacter* sp.	KJ152098.1	*Gammaproteobacteria*	99%
M483	*Rahnella* sp.	KF720928.1	*Gammaproteobacteria*	99%
M507	*Ewingella* sp.	KX378906.1	*Gammaproteobacteria*	99%
M508	*Rahnella bruchi*	NR_146845.1	*Gammaproteobacteria*	99%
M511	*Rahnella bruchi*	KF308407.1	*Gammaproteobacteria*	98%
M513	*Enterobacter ludwigii*	JF505955.1	*Gammaproteobacteria*	99%
